# Causative factors and rehabilitation of patellar tendinopathy: A systematic review

**DOI:** 10.4102/sajp.v72i1.338

**Published:** 2016-11-29

**Authors:** Sanell Morgan, Elizabeth C. Janse van Vuuren, Frederik F. Coetzee

**Affiliations:** 1 Department of Physiotherapy, University of the Free State, South Africa; 2 Department of Exercise and Sport Sciences, University of the Free State, South Africa

## Abstract

**Background:**

Patellar tendinopathy (PT) is a common chronic pathology of the knee, with a high prevalence in athletes and the general population.

**Objectives:**

The objectives of this article were to systematically investigate all the evidence applicable to the intrinsic and extrinsic causative factors and rehabilitation of PT, and then integrate and link rehabilitation with the main causative factors identified.

**Method:**

The Preferred Reporting Items for Systematic Reviews and Meta-Analyses guidelines were followed. Various tools were used to evaluate the methodological quality of the eligible articles. Data were interpreted descriptively, and the causative factors and rehabilitation of PT were analysed.

**Results:**

Twenty studies were included in the review. The distinctive factor responsible for PT is the mechanical theory. Seven intrinsic and four extrinsic risk factors were identified, with the main intrinsic risk factors being muscle flexibility and strength, and extrinsic risk factors being acquisition and level of skills. PT can be treated with numerous different therapeutic modalities, although eccentric muscle training showed exceptional results. The intrinsic and extrinsic risk factors can only be transformed and reduced by rehabilitation, which is inevitable to improve PT pain and function.

**Conclusion:**

The essence of an integrated management protocol for PT is to identify the dominant contributing factors, whether intrinsic or extrinsic, and to reduce the load on the patellar tendon by modifying these factors by either rehabilitation intervention or direct modification of the equipment or environment to obtain a positive outcome towards pain management and function.

## Introduction

Patellar tendinopathy (PT) is a common chronic pathology of the knee, with a high prevalence in both athletes (Frizziero *et al*. 2014) and the general population (Toppi *et al*. 2015). It is characterised by microscopic tears and tissue degeneration because of excessive and repetitive mechanical loading of the patellar tendon. Epidemiological studies indicate that tendon injuries account for possibly up to 50% of injuries sustained during sporting activities, with tendon overuse because of running-associated sports accounting for nearly 30%. Athletes with PT can experience uncomfortable symptoms and decreased function for up to 3 years (Saggini *et al*. 2012), which have a negative effect on quality of life (Toppi *et al*. 2015). The risk factors for and treatment of PT can be challenging and unsatisfying (Silva *et al*. 2015). A number of risk factors for the development of PT have been identified, such as age, gender, heavy physical work, type of training surface, high training volume and level of participation (De Vries *et al*. 2015a). A better understanding of the aetiology of PT will facilitate the identification of modifiable risk factors and make a valuable contribution to planning of preventative measures and interventions (Van der Worp *et al*. 2012). It is unclear how these risk factors specifically relate to the available rehabilitation options for PT.

Only one published systematic review article (Van der Worp *et al*. 2011b) indicated the risk factors for PT, whereas three others described different rehabilitation options for PT (Frizziero *et al*. 2014; Malliaras *et al*. 2013; Mani-Babu *et al*. 2015). However, none of these reviews integrated the risk factors and rehabilitation. One of the objectives of this review was therefore to integrate the risk factors and rehabilitation of PT in a systematic review. This followed a methodical investigation of all evidence applicable to the intrinsic and extrinsic causative factors as well as rehabilitation of PT, as another objective of this review. Addressing the intrinsic and extrinsic factors with rehabilitation is considered to be a good measure in the successful rehabilitation of PT, and therefore, this systematic review provides a unique and valuable perspective for healthcare professionals involved in the management of PT, by integrating the causative factors and rehabilitation of PT.

## Research design

### Research method

The Preferred Reporting Items for Systematic Reviews and Meta-Analyses (PRISMA) guidelines were used for the systematic review (Moher *et al*. 2009). Published articles were considered based on the inclusion and exclusion criteria listed in Box 1.

BOX 1 Inclusion and exclusion criteria.

**Inclusion criteria**

**Publication period:** Articles published between January 2010 and October 2015

**Research design:** Systematic reviews, randomised clinical trials, non-randomised clinical trials, quantitative research studies, qualitative research studies

**Age of participants:** Between 18 and 60 years

**Research focus:** Causative factors (intrinsic and extrinsic) and/or rehabilitation of patellar tendinopathy (PT)

**Exclusion criteria**

**Population:** Participants with other knee pathologies, previous knee surgery or injection therapy in the knee

**Research focus:** Patellar tendinopathy (PT), but excluding causative factors and/or rehabilitation

**Language of article:** Articles published in any other language than English

**Availability of article:** Articles of which only the abstract was available

### Search strategy

Electronic databases available on EBSCOhost were searched and included Academic Search Complete, Africa-Wide Information, MEDLINE with Full Text, AHFS Consumer Medication Information, CINAHL with Full Text, ERIC, Health Source – Consumer Edition, Health Source: Nursing/Academic Edition, Humanities Source, PsycARTICLES, PsycEXTRA, PsycINFO, PsycTESTS, SocINDEX with Full Text, SPORTDiscus with Full Text. The search was conducted by the authors and one independent researcher (‘research team’ hereafter), for articles published between January 2010 and October 2015. This specific time period was selected, as this review aimed to follow up on a previous systematic review conducted by Van der Worp *et al*. (2011b) on PT that included articles up to August 2010.

The search strategy included the following keywords in order to identify all the relevant articles for inclusion in this review:

(“patella* tendinopath*” or (patella* and tendinit*))

AND

(“intrinsic factor*” or age or gender or “body composition*” or “fat mass” or “body weight” or “body mass index” or injur* or “joint instability*” or “musc* strength” or “musc* power” or “range of motion” or “range of movement” or “anatomic* alignment*” or “postural stability*” or “sport* specific technique*” or “level of skill*” or “skill* level*” or “extrinsic factor*” or strapping or bracing or “foot wear” or footwear* or shoe* or “training surface*” or “eccentric decline squat*” or “skill* acquisition” or proprioception* or flexib* or “muscle activat*” or etiolog* or aetiolog*)

AND

(rehab* or “return to sport” or “return to play” or “motor re-educat*”) and (exercise* or train* or sport*)

**FIGURE 1:**
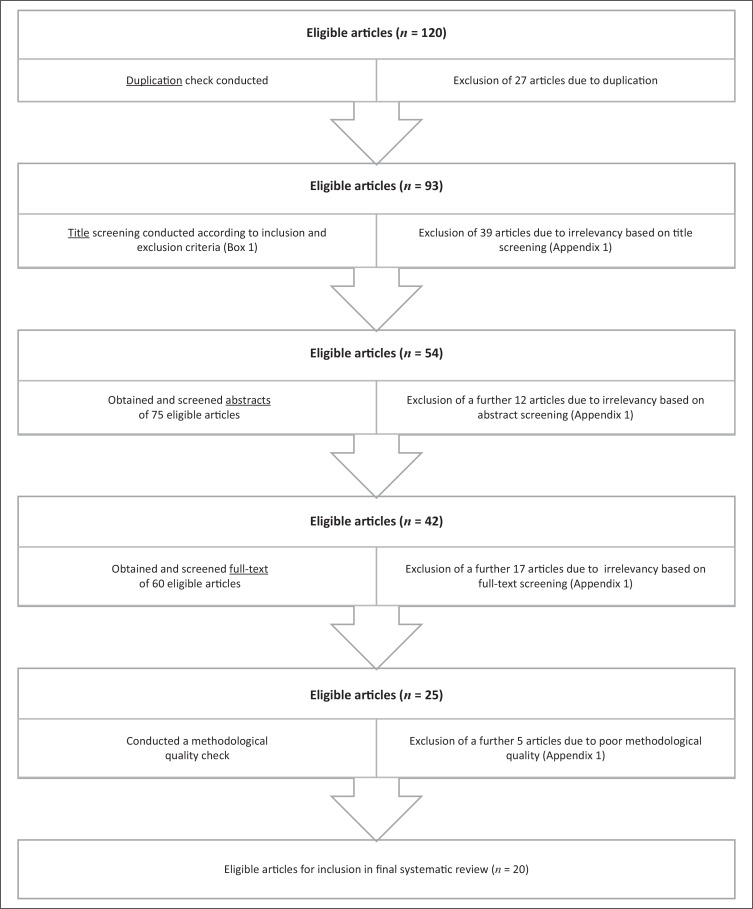
Flow diagram of search strategy to determine the final sample of the articles for the review.

This search was conducted twice during the study period (approximately 3 months apart), in order to cross-reference the results and ensure that all possible articles eligible for inclusion were identified. The combination of results from the two searches yielded 120 possible articles for inclusion before the elimination of duplicate studies (Figure 1).

### Study selection

The study selection process from the eligible 120 articles was independently conducted by all members of the research team, against the inclusion and exclusion criteria (Box 1). When there was vagueness concerning the eligibility, a conversation was held between all the members of the research team to resolve any disagreement. Figure 1 shows the process of the search strategy to determine the final sampling of the articles for the review.

## Methodology quality appraisal

Different methodology quality appraisal tools were selected to evaluate the eligible articles because of the different research designs of these articles. Twenty-five articles were assessed for methodology quality scoring after which 20 articles were included in this systematic review (see Figure 1). Table 1 lists more details of the methodological quality appraisal for the final 20 articles included in this systematic review. The independent researcher was responsible for verifying the data obtained from the scoring done by the authors. The average total methodological quality score for all the articles included (*n* = 20) was 72%.

**TABLE 1:** Methodology assessment tools.

Study design	Assessment tools	Quality scoring range	Average quality scoring	Reasons for exclusion
Systematic reviews	AMSTAR Checklist	6/11–10/11 (*n* = 4)	71% (*n* = 4)	No *priori* design No duplicate study selection and data extraction At least two electronic sources searched No status of publication used as an inclusion checklist No list of studies provided, no characteristics of included studies No scientific quality of articles Scientific quality not used in formulating conclusions No appropriate methods to combine findings No publication bias assessed No conflict of interest stated
Randomised clinical trials	PEDro Scale	8/11–9/11 (*n* = 3)	76% ( *n*= 3)	Less than 50% on methodology quality scoring
Non-randomised clinical trials	Downs & Black Checklist	16/27–20/27 (*n* = 3)	68% (*n* = 3)	Less than 50% on methodology quality scoring
Qualitative research	Methodology Checklist – Qualitative	10/14–13/14 (*n* = 5)	82% (*n* = 5)	Less than 50% on methodology quality scoring
Quantitative research	National Institute for Health and Excellence Checklist	15/27–21/27 (*n* = 5)	63% (*n* = 5)	Less than 50% on methodology quality scoring

### Data extraction

A custom-made Excel spreadsheet was developed to extract applicable information from each article and a summary is displayed in Tables 2, 3 and 4. Information was documented with regard to the age, gender, body composition, muscle strength and flexibility, anatomical alignment, medical history, strapping or orthosis, training surfaces, and acquisition and level of skill of the participants described in each article. The researcher conducted the data extraction autonomously. The information recorded was confirmed by the rest of the research team who checked it jointly for mistakes after completion of the process.

**TABLE 2:** The intrinsic and extrinsic causative factors for PT in the articles included in the review.

Authors (date)	Study design	Study participants	Causative intrinsic factors	Causative extrinsic factors
De Vries *et al*. (2015a)	Survey-based prospective cohort study	**Age:** 18–35 years **Gender:** Male and female **Level of skills:** Non-elite and elite volleyball and basketball players	**Gender:** Male > female	**Acquisition of skills:** Heavy physical work
De Vries *et al*. (2015b)	Randomised clinical trail	**Age:**18–50 years **Gender:** Male > female	*Not included*	**Strapping or orthosis:** ↓ PT pain, ↑ proprioception, female > male ↑ outcome
Toppi *et al*. (2015)	Prospective cohort study	**Age:**40–67 years **Gender:** Female	**Muscle strength:** Larger vastus medialis muscle	**Level of skills:** Higher levels of physical activity
Van der Worp *et al*. (2012)	Cross-sectional study	**Age:**18–35 years. **Gender:** Male and female	**Age:**18–35 years; decreased risk for PT with increasing age **Gender:** Male > female for PT	**Level of skills:** Higher level of participation is a risk factor for PT **Acquisition of skills:** Some volleyball positions are more likely to develop PT **Type of sport:** Volleyball > basketball
Van der Worp *et al*. (2011b)	Systematic review	*Not included*	**Body composition:** Weight, body mass index, waist-to-hip ratio **Anatomical alignment:** Leg-length discrepancies, arch height of the foot **Muscle flexibility:** Decreased quadriceps, hamstring flexibility **Muscle strength:** Decreased quadriceps strength and vertical jump performance	*Not included*
Van der Worp *et al*. (2011a)	Online survey: descriptive	**Age:**18–35 years **Gender:** Male and female	**Gender:** Twice as high in males than in females	**Acquisition of skills:** Heavy physical work
Souza *et al*. (2010)	Experimental study	**Age:** Experimental group mean 28.9 years; control group mean 24.9 years **Gender:** Male	**Anatomical alignment:** ↑ hip extensor contribution and ↓ knee extensor contribution in total support moment	*Not included*

**TABLE 3:** Rehabilitation of patellar tendinopathy.

Authors (year)	Study design	Study participants	Rehabilitation
Mani-Babu *et al*. (2015)	Systematic review and meta-analysis	*Not included*	Extracorporeal shock wave therapy
Frizziero *et al*. (2014)	Review and meta-analysis	*Not included*	Eccentric training
McCreesh, Riley and Crotty (2013)	Case report	**Age:**34 years **Gender:** Male	Eight-week programme of eccentric training, twice/day, 10 repetitions
Malliaras *et al*. (2013)	Systematic review	*Not included*	Eccentric training; eccentric-concentric loading alongside
Dimitrios, Pantelis and Kalliopi (2012)	Controlled clinical trial	**Age:**18–30 years **Gender:**16 male, 6 female	Eccentric training as well as eccentric training and static stretching in combination
Da Cunha *et al*. (2012)	Randomised controlled study	**Age:**18 years **Gender:**14 male, 3 female	Eccentric training**:** 12 weeks with or without pain
Van der Worp *et al*. (2011c)	Randomised controlled trial	**Age:**18–50 years	Focus and radial shockwave therapy, three times per week with eccentric training, 2 × 15, twice daily, 5 days a week
Romero-Rodriguez, Gual and Tesch (2011)	Case series	**Age:**18–35 years **Gender:** Male	Eccentric training**:** 6 weeks, 12 sessions for 24 minutes
Zwerver *et al*. (2010)	Pilot study	**Age:**18–58 **Gender:** Male	Three extracorporeal shock wave therapy treatments
Goldman and Lentz (2010)	Case report	**Age:**18 years **Gender:** Male	Eccentric training: 6 weeks, once daily, three times per week, stretching and strengthening

**TABLE 4:** Causative factors and rehabilitation.

Authors (year)	Study design	Participants	Causative intrinsic factors	Causative extrinsic factors	Rehabilitation
Silva *et al*. (2015)	Case report	**Age:**21 years **Gender:** Male	**Muscle flexibility:** Decreased flexibility quadriceps, hamstrings **Muscle strength:** Decreased muscle strength of quadriceps **Range of motion:** Decreased dorsiflexion **Anatomical alignment:** Longitudinal arch height of the foot	*Not included*	Eight weeks, 3 times per week, 30 minutes. Gluteus maximus strengthening bilateral 3 × 15. Drop jumps 3 × 10. Resume sport participation < 3/10 on the Visual Analogue Scale
Saggini *et al*. (2012)	Prospective, single-centre study	**Age:**18–34 years **Gender:** Male	**Anatomical alignment:** Lower patella pole	**Level of skills: >** 12 hours per week of training/playing, weight training > 5 hours per week **Training surfaces:** Hard surfaces	Three weeks, one session of extracorporeal shock wave therapy and three physiotherapy sessions of eccentric training per week
Samukawa (2011)	Case review	**Age:**29 years **Gender:** Female	**Anatomical alignment:** Frog’s eye patellae bilaterally, patella baja right patella-femoral joint, hips external alignment, 1 cm left > right leg, Q angle of 10° both legs **Muscle flexibility:** Ober test + bilateral, Thomas test +, J-sign bilateral **Range of motion:** Decreased internal hip rotation and dorsiflexion bilateral	*Not included*	Eight weeks, 15-minute jog, static stretching lower limb, eccentric training, closed-chain strengthening of quadriceps, cryotherapy and proprioception

### Data analysis

Pooling of the data for the purpose of framing a meta-analysis was not an aim of the systematic review because of dissimilarities in the outcomes. Data were summarised descriptively by means of gathering information about the characteristics of the causative factors and rehabilitation of PT to form a data analysis.

## Results

The search strategy yielded 20 articles that met the described inclusion criteria shown in Box 1. Results from these 20 articles are included and discussed in the next section.

### Demographic information

The age of the study participants varied for each of the 15 studies in this review that revealed the age of the participants (see Tables 2, 3 and 4). All the study participants were older than 18 years, with Toppi *et al*. (2015) having the oldest study participants (with a mean age of 67 years). The majority of the study participants were aged between 18 and 35 years. According to Van der Worp *et al*. (2012), the probability of sustaining PT decreased with age, but they argued that the risk for PT increased for individuals older than 30 years of age because of changes in the tendon structure and its mechanical properties. However, the correlation between PT and age is still uncertain (Van der Worp *et al*. 2012).

Fourteen of the studies included in this review (Tables 2, 3 and 4) described gender with 13 indicating that the majority of participants were men. Three of these studies determined gender to be a risk factor, with men more likely to develop PT than women (De Vries *et al*. 2015a; Van der Worp *et al*. 2011a, 2012). This hypothesis could be justified in that the quadriceps muscle generates more force during contraction in male than in female athletes, as well as the fact that women’s oestrogen plays a role in influencing the tendon structures positively (Van der Worp *et al*. 2012). Nevertheless, PT is a multifactorial pathology and it is challenging to obtain direct evidence with regard to gender differences and the part played by oestrogen (Van der Worp *et al.* 2011a).

### Intrinsic causative factors

#### Muscle flexibility

According to the three systematic reviews, impaired lower limb muscle flexibility was noted as being a risk factor for PT. The muscles described in these reviews are the iliotibial band (ITB) (Samukawa 2011), quadriceps and hamstring muscles (Silva *et al*. 2015; Van der Worp *et al*. 2011b). Stiffness in the ITB can cause lateral patella movement because of its anatomical attachments and can also contribute to decreased lower limb flexibility involving the quadriceps and hamstring muscles (Samukawa 2011). Impaired quadriceps and hamstring muscle flexibility intensify tendon strain during joint movement, leading to tendon overload and the development of PT (Van der Worp *et al*. 2011b).

#### Muscle strength

Three of the studies raised the argument that muscle strength could be associated with PT. Both greater vastus medialis muscle strength (Toppi *et al*. 2015) and quadriceps muscle atrophy have been identified as risk factors for PT (Silva *et al*. 2015; Van der Worp *et al*. 2011b).

#### Anatomical alignment

Four studies referred to anatomical alignment in athletes with PT. Saggini *et al*. (2012) reported a definite correlation between an inferior patellar pole alignment and the probability to develop PT. Secondly, leg-length discrepancy, where the longer leg is the preferred take-off leg in jumping, can also be associated with PT. However, there is limited evidence to confirm this (Van der Worp *et al*. 2011b). Another hypothesis is that lower foot arch heights might cause knee and soft tissue injuries (Silva *et al*. 2015). It is anticipated that greater quadriceps muscle contraction is desired to avoid further knee flexion (Van der Worpe *et al*. 2011b).

#### Body composition

Three studies mentioned body mass index (BMI), with two of them not finding any relationship between BMI and PT (De Vries *et al*. 2015a; Toppi *et al*. 2015). The third study indicated that a higher BMI can contribute to PT because of a theoretically greater loading of the patellar tendon (Van der Worp *et al*. 2011b).

#### Joint range of movement

Samukawa (2011) and Silva *et al.* (2015) mentioned in both their case reports that decreased dorsiflexion was a risk factor for PT. This must, however, be interpreted with caution because of the methodology used (i.e. only one study participant per case report).

### Extrinsic causative factors

#### Acquisition and level of skills

In sporting activities where the level and acquisition of skills are important, the development of PT is a possibility, as noticed in five studies included in this review (Saggini *et al*. 2012; Samukawa 2011; Souza *et al*. 2010; Toppi *et al*. 2015; Van der Worp *et al*. 2012). Research also indicated a direct link between higher levels of participation in sport (Van der Worp *et al*. 2012) and physical activity (Toppi *et al*. 2015) as risk factors for PT. Additionally, some possible supplementary extrinsic risk factors that could influence the development of PT are player position, an increased demand in training hours per week, number of games per month, increased training from year to year, the amount of hours participating in additional sports (Van der Worp *et al*. 2012) and weight training for at least 5 hours per week (Saggini *et al*. 2012). Heavy physical work has a considerable effect on the development of PT in sports-related and non-sports-related basketball and volleyball players, which affects their work performance (De Vries *et al*. 2015a; Van der Worp *et al*. 2011a).

#### Type of sport

Only one article referred to the type of sport as an extrinsic causative risk factor for PT. Volleyball players are more likely to develop PT than basketball players, possibly because of the difference in the number of jumps performed and the players’ jumping technique (Van der Worp *et al*. 2012).

#### Training surface

Saggini *et al*. (2010) were the only authors who reported that training on hard surfaces is a risk for PT because of the load on the tendon. It has been suggested that softer training surfaces may reduce the risk (Van der Worp *et al*. 2012).

#### Strapping or orthosis

According to De Vries *et al*. (2015b), a patella strap or sports tape decreases pain in an experimental group compared with a control group in the short term, although none of them is more effective than placebo taping. The long-term effect remains unclear, with an amplified effectiveness of taping in female participants (De Vries *et al*. 2015b).

### Rehabilitation

Thirteen of the 20 studies included in the systematic review had one or more component that described parts of the rehabilitation intervention for PT. The literature reported on rehabilitation intervention comprising cryotherapy (Samukawa 2011), lower limb strengthening (Frizziero *et al*. 2014; Goldman & Lentz 2010; Malliaras *et al*. 2013), lower limb stretching (Goldman & Lentz 2010) and proprioception retraining (Samukawa 2011). Table 3 summarises the rehabilitation for PT.

#### Eccentric exercise

Eccentric exercise (EE) is extremely popular in the conventional treatment of chronic lower limb tendinopathies (Saggini *et al*. 2012), and literature on EE dates back to 1938. It is therefore not surprising that 10 of the studies included for review described EE as a rehabilitation modality for PT. McCreesh *et al*. (2013) clarified that the mechanism of action of EE is to resolve the neovascularity in the patellar tendon.

Positive rehabilitation intervention results can also be accomplished by combining EE with other treatment modalities, such as extracorporeal shock wave therapy (ESWT) (Saggini *et al*. 2012) and static stretches of the lower limb (Dimitrios *et al*. 2012). Four studies reported that ESWT (Mani-Babu *et al*. 2015) is a promising adjunctive (Zwerver *et al*. 2010) modality for PT in both short- and long-term programmes (Frizziero *et al*. 2014; Mani-Babu *et al*. 2015). Malliaras *et al*. (2013) suggested that an eccentric-concentric loading programme for individuals with PT is more beneficial than EE on its own, particularly in athletes with noticeable concentric muscle weakness that may not recover with isolated EE because of the muscle type contraction.

#### PT and other treatment modalities

Non-specified drug therapy (Dimitrios *et al*. 2012) and deep transverse friction massage also improve the symptoms of PT (Samukawa 2011).

#### Hip and core strengthening

The gluteus maximus muscle is considered to be an important hip extensor muscle to strengthen during PT rehabilitation (Silva *et al*. 2015).

#### Stretches

Improved flexibility of the muscles of the lower limb, especially muscles surrounding the hip and knee, forms an essential component of rehabilitation and the resolution of chronic symptoms of PT (Samukawa 2011).

#### Return to sport

There are contradicting views regarding return to sport during the rehabilitation intervention. Silva *et al*. (2015) advised that sport participation can be continued, but the pain experienced during the activity may not exceed 3/10 on the Visual Analogue Scale. On the contrary, it is recommended that activity participation must only resume after tendon healing is complete (Dimitrios *et al*. 2012), which is suggested to take approximately 12 weeks (Frizziero *et al*. 2014). Sport-specific activities that cause pain in the tendon must be avoided until the tendon has healed completely (Dimitrios *et al*. 2012).

#### Sport-specific technique

Jumping with appropriate kinematics is an important factor to consider in PT. The jumps in PT athletes must be evaluated and modified accordingly to produce a ‘soft landing’ to reduce the load on the tendon (Silva *et al*. 2015).

## Discussion

PT is a diverse and complex pathology with numerous challenges regarding risk factors for developing PT and its rehabilitation, and it can be more complicated than assumed to identify these aspects. A limited number of articles (*n* = 20) were included in the systematic review because of the inclusion and exclusion criteria for this review, as well as poor methodological quality of a number of studies initially identified. This, in addition to the heterogeneity of the studies when considering specific aspects related to the causative factors and rehabilitation, prevented a meta-analysis to be performed.

### Intrinsic and extrinsic causative risk factors for patellar tendinopathy

The initial aim of this systematic review was to review the literature concerning the intrinsic and extrinsic causative risk factors for PT. The distinctive factor responsible for the development of PT is the mechanical theory. It can be defined as a failed healing process with micro-injuries to the patellar tendon because of overloading, which is responsible for matrix and cell changes and altered mechanical properties of the tendon (Van der Worp *et al*. 2011b). The data varied and seven intrinsic and four extrinsic risk factors were identified. The main intrinsic risk factors identified were muscle flexibility and strength. Regarding the extrinsic risk factors, acquisition and level of skills were the prominent factors. Evidence for all the other risk factors was indecisive. Although several risk factors for PT were recognised, the systematic review did not demonstrate robust evidence for numerous intrinsic or extrinsic causative risk factors, which has possibly been limited by the inclusion and exclusion criteria for this review.

If the mechanical pathophysiological theory discussed previously (Van der Worp *et al*. 2011b) is taken in consideration, it clearly indicates that all intrinsic and extrinsic causative risk factors for PT mentioned in the results are responsible for loading the patellar tendon in altered ways (see intrinsic causative risk factors and Table 2). If the intrinsic and extrinsic causative factors could be addressed in rehabilitation and the load on the tendon could be minimised, improvement in pain and function will be noticeable.

### Rehabilitation for patellar tendinopathy

Regarding the rehabilitation, mainly descriptive articles with a widespread range of research designs were included. PT can be treated with numerous different therapeutic modalities; EE training showed exceptional results, with 10 of the 13 articles on rehabilitation for PT reporting positive results. The perception is still that EE is the gold standard conservative treatment modality and superior to other treatment options based on its excellent and sturdy outcomes over the years. This happens when EE lengthens the tendon, which is responsible for ‘squeezing out’ the flow of neovessels (McCreesh *et al*. 2013). Doppler ultrasound imaging after a period of EE indicated a minimal improvement in tendon echogenicity and a substantial decline in tendon neovascularity, with a dramatic improvement in the symptoms and pain in the tendon (Da Cunha *et al*. 2012; Samukawa 2011). This explains why EE is so successful and beneficial in the treatment of PT. EE not only assists with diminishing of neovascularity, but also facilitates improvements in neuromuscular activation, improved muscle strength (Samukawa 2011) and muscle endurance (Saggini *et al*. 2012).

EE is performed as a single leg squat on a decline board with an angle of 25° at a slow speed to assist with tissue healing (Dimitrios *et al*. 2012) and has superior results over a squat that is performed on a flat-step (Frizziero *et al*. 2010; Goldman & Lentz 2010). EE can be performed in an aggressive manner causing pain (Da Cunha *et al*. 2012) or without pain, because evidence reveals that both establish improvements in pain and function (Da Cunha *et al*. 2012). The frequency and repetitions of performing EE differed in the reviewed articles, but generally included three sets of 15, one or twice daily and 5–7 days a week (Goldman & Lentz 2010; McCreesh *et al*. 2013; Van der Worp *et al*. 2011c).

EE can be performed at home without full-time supervision, although the athlete’s compliance may influence the outcomes (Dimitrios *et al*. 2012). The high intensity of performance of EE seems to be decisive for favourable therapeutic results (Da Cunha *et al*. 2012). More than one of the articles included in this review combined EE with an additional treatment modality. Although this systematic review did not specifically aim to address the possible different treatment methods in combination with EE, it has been noticed that EE has positive outcomes when used in combination with other treatment modalities.

Multiple treatment options are available for PT, and the collective aim is to improve pain and function in PT. One of these additional modalities is ESWT, of which the therapeutic outcome is based on its bio-stimulating effects (Saggini *et al*. 2012). The authors suggested different dosage prescriptions which vary from one (Saggini *et al*. 2012) to three treatment sessions per week for 3 weeks (Van der Worp *et al*. 2011c). Secondly, static stretches contribute to the reversal of PT (Dimitrios *et al*. 2012), and deep transverse friction massage contributes to reduce the adhesions and enable realignment of collagen fibres (Samukawa 2011).

An important factor in managing symptoms of tendinopathy during the rehabilitation intervention is regulating or reducing the tendon load for good execution of the exercise programme.

Romero-Rodriguez *et al*. (2011) described a rehabilitation programme with training twice a week for 24 minutes per session, whereas Silva *et al.* (2015) suggested the frequency to be three times a week with a duration of 30 minutes per session. The length of the rehabilitation period varied from 6 to 12 weeks, as proposed by different authors (Da Cunha *et al*. 2012; Goldman & Lentz 2010). Romero-Rodriguez *et al*. (2011) proposed a rehabilitation period of 6 weeks to have positive results in highly trained athletes, although a prolonged rehabilitation period may be applicable because of the sluggish recovery of the patellar tendon (Saggini *et al*. 2012). The benefits of a conservative rehabilitation programme can include an improvement in knee range of movement, quadriceps muscle strength, reduction in PT pain symptoms and general improvement in knee function (Goldman & Lentz 2010).

### The association between intrinsic and extrinsic causative risk factors with rehabilitation

Intrinsic and extrinsic causative risk factors can only be addressed by rehabilitation which is essential to the improvement of PT pain and function. Therefore, it is essential to address each intrinsic and extrinsic causative risk factor individually during the rehabilitation period and make adaptations to the environment if necessary. Research studies describing the rehabilitation of intrinsic and extrinsic causative factors were found in the results of this systematic review (see Tables 2, 3 and 4).

A positive observation from this systematic review is that inadequate lower limb flexibility and muscle strength, especially around the hip and knee, has an adverse effect on knee kinematics (Samukawa 2011) and can be addressed by a stretching and strengthening programme. It is advised to start with non-weight bearing strengthening exercises and then progress to more advanced exercises that will contribute to improve lower limb biomechanics during landing kinematics (Silva *et al*. 2015). This indicates a direct link between addressing intrinsic and extrinsic causative factors in rehabilitation and the difference in the pain and function associated with PT.

Acquisition and level of skills are extrinsic risk factors for PT that can be modified during rehabilitation to correct the execution technique of activities and lower the demand on the tendon. For example, in a task requiring lower limb effort, such as hopping, it has been found that athletes with PT perform the task differently to ‘off-load’ the knee and decrease the effort on the affected joint (Souza *et al*. 2010). This requires evaluation of the athlete’s technique and the necessary adjustments must be made. De-loading of the tendon can also be achieved by means of decreasing the load by reducing the frequency, intensity and duration of activities (Van der Worp *et al*. 2012). Furthermore, working activities must be considered when modifying the load on the tendon (Van der Worp *et al*. 2011a), and load progression must always be done gradually (Goldman & Lentz 2010). Prevention of PT can be promoted by taking note of complaints and monitoring athletes individually to identify any symptoms of PT (Van der Worp *et al*. 2012).

Some recommendations regarding the causative factors and rehabilitation are relatively broad rather than explicit guidelines. Therefore, there is a need for continuous high-quality research studies in order to identify better evidence regarding the intrinsic and extrinsic risk factors associated with PT and to investigate the diverse rehabilitation interventions for tailoring a rehabilitation programme to manage this challenging pathology.

A limitation of this systematic review was the inclusion of a limited number of articles because of the specific research focus, inclusion and exclusion criteria set for this review, and the poor methodological quality of a number of studies initially identified. In an attempt to address this limitation, the researchers applied the search strategy twice during the study period and also examined the reference lists of all the included studies in an effort to identify possible additional studies. This additional measure, however, yielded no further articles for inclusion.

Strengths of this systematic review include the perspectives presented, which give insight into the causative factors and rehabilitation for PT. This will enhance the knowledge of health care professionals involved in the management of PT. Another strength is the inclusion of articles with a good methodological quality (i.e. with an average of 72%) following the rigorous methodology quality assessment. The results, however, indicate a high incidence of similar authors included in the systematic review, which may direct publication bias. PT is a troublesome pathology which requires intensive research and rehabilitation and the high methodological quality of articles included in this review contribute to reliable outcomes in the reporting of the findings.

## Conclusion

This systematic review presents a unique perspective on the integration of intrinsic and eccentric causative factors with the rehabilitation of PT. This insight into the latest evidence highlights the essence of an integrated management protocol for PT to obtain a positive outcome with regard to pain management and function in athletes with PT.

## References excluded articles

### Appendix 1

#### Exclusion of articles because of irrelevancy based on title screening (*n* = 39)

Abat, F., Diesel, W.J., Gelber, P.E., Polidori, F., Monllau, J.C., Sanchez-Ibanez, J.M. 2014. Effectiveness of the intratissue percutaneous electrolysis (EPI) technique and isoinertial eccentric exercise in the treatment of patellar tendinopathy at two years follow-up. *Muscles, Ligaments and Tendons Journal* 4(2):188–193.

Abstracts of the International Federation of Associations of Anatomists 2014. http://www.csas.org.cn/ifaa2014/AANAT.pdf Retrieved 12 September 2016.

Biernat, R., Trzaskoma, Z., Trzaskoma, L., Czaprowski, D. 2014. Rehabilitation protocol for patellar tendinopathy applied among 16- to 19-year old volleyball players. *Journal of Strength and Conditioning Research* 28(1):43–52.

Brockmeyer, M., Diehl, N., Schmitt, C., Kohn, D.M., Lorbach, O. 2015 Results of surgical treatment of chronic patellar tendinosis (jumper’s knee): A systematic review of the literature. *The Journal of Arthroscopic & Related Surgery* 31(12):2424–2429.

Camargo, P.R, Alburquerque-Sendin, F., Salvini, T.F. 2014. Eccentric training as a new approach for rotator cuff tendinopathy: Review and perspectives. *World Journal of Orthopedics* 5(5):634–644.

Campbell, R.S.D., Dunn, A.J. 2012. Radiological interventions for soft tissue injuries in sport. *British Journal of Radiology* 85(1016):1186–1193.

Clark, N.T.M., Bourque, R.D., Schilling, J., McKeon, P. Treatment of patello-femoral pain syndrome in a track athlete. *International Journal of Athletic Therapy & Training* 19(1):27–31.

Ellenbecker, T.S., Pieczynski, T.E., Davies, G.J. 2010. Rehabilitation of the elbow following sports injury. *Clinics in Sports Medicine* 29(1):33.

Filardo, G., Kon, E., Di Matteo, B., Di Martino, A., Tesei, G., Pelotti, P., Cenacchi, A., Marcacci, M. 2014. Platelet-rich plasma injections for the treatment of refractory Achilles tendinopathy: results at 4 years. *Blood Transfusion* 12(4):533–540.

Filardo, G., Kon, E., Di Matteo, B., Pelotti, P., Di Martino, A., Marcacci, M. 2013. Platelet-rich plasma for the treatment of patellar tendinopathy: clinical and imaging findings at medium-term follow-up. *International Orthopaedics* 37(8):1583–1589.

Gill, T.J., Carroll, K.M., Hariri, S. 2013. Open patellar tendon debridement for treatment of recalcitrant patellar tendinopathy: Indications, technique, and clinical outcomes after a 2-year minimum follow-up. *Sports Health:* *A Multidisciplinary Approach* 5(3):276.

Hadzic, V., Sattler, T., Markovic, G., Veselko, M., Dervisevic, E. 2010. The isokinetic strength profile of quadriceps and hamstrings in elite volleyball players. *Isokinetics and Exercise Science* 18(1):31–37.

Hall, R., Barber Foss, K., Hewett, T.E., Myer, G.D. 2015. Sport specialization’s association with an increased risk of developing anterior knee pain in adolescent female athletes. *Journal of Sport Rehabilitation* 24(1):31–35.

Hernandez, V.P., Varela, S.M., Moraleda, B.R. 2011. Proposal for functional recovery from ruptured anterior cruciate ligament in soccer. *Revista Internacional de Medicina y Ciencias de la Actividad Fisica y del Deporte* 11(43):573–591.

Kaux, J. F., Croisier, J. L., Bruyère, O., Rodriguez, C., Daniel, C., Godon, B., Simoni, P., Alvarez, V., Brabant, G., Lapraille, S., Lonneux, V., Noël, D., Collette, J., Le Goff, C., Gothot, A., Crielaard, J. M. 2013. Platelet-rich plasma (PRP) to treat chronic upper patellar tendinopathies. *British Journal of Sports Medicine* 47(10):6.

Kaux, J.F., Croisier, J.L., Forthomme, B., Le Goff, C., Delcour, S., Gothot, A., Crielaard, J.M. 2015. Exploring the effect of a second closely-timed infiltration of platelet-rich plasma to treat jumper’s knees. *Annals of Physical & Rehabilitation Medicine* 58:68.

Kaux, J.F., Croisier, J.L., Bruyere, O. Rodriguez De La Cruz, C., Forthomme, B., Brabant, G., Lapraille, S., Lonneux, V., Noel, D., Le Goff, C., Gothot, A., Collette, J., Crielaard, J.M. 2015. One injection of platelet-rich plasma associated to a submaximal eccentric protocol to treat chronic jumper’s knee. *The Journal of Sports Medicine and Physical Fitness* 55(9):953–961.

Kaux, J.-F., Forthomme, B., Namurois, M-H, Bauvir, P., Defawe, N., Delvaux, F., Lehance, C., Crielaard, J.-M., Croisier, J.-L. 2014. Description of a standardized rehabilitation program based on sub-maximal eccentric following a platelet-rich plasma infiltration for jumper’s knee. *Muscles, Ligaments & Tendons Journal* 4(1):85–89.

Kulig, K., Noceti-DeWit, L.M., Reischl, S.F., Landel, R.F. 2015. Physical therapists’ role in prevention and management of patellar tendinopathy injuries in youth, collegiate, and middle-aged indoor volleyball athletes. *Brazilian Journal of Physical Therapy* 19(5):410–420.

Liddle, A.D., Rodríguez-Merchán, E.C. 2015. Platelet-rich plasma in the treatment of patellar tendinopathy. *American Journal of Sports Medicine* 43(10):2583–2590.

Maffulli N., Del Buono A., Oliva F., Testa V., Capasso G., Maffulli, G. 2015. High-volume image-guided injection for recalcitrant patellar tendinopathy in athletes. *Clinical Journal of Sport Medicine*:26(1):12–16.

Maier, D., Bornebusch, L., Salzmann, G.M., Sudkamp, N.P., Ogon, P. 2013. Mid- and long-term efficacy of the arthroscopic patellar release for treatment of patellar tendinopathy unresponsive to nonoperative management. *Arthroscopy-The* *Journal of Arthroscopic and Related Surgery* 28(8):1338–1345.

Marcheggiani M., Giulio M., Zaffagnini, S., Tsapralis, K., Alessandrini, E., Bonanzinga, T., Grassi, A., Bragonzoni, L., Della Villa, S., Marcacci, M. 2013. Open versus arthroscopic surgical treatment of chronic proximal patellar tendinopathy. A systematic review. *Knee Surgery, Sports Traumatology, Arthroscopy* 21(2): 351–357.

Martínez-Rodríguez, A., Moreno-Pérez, V., Roche, E., Vicente-Salar, N. 2013. Planificación dietética y rehabilitación a Largo Plazo de Jugadores profesionales de tenis y fútbol mediante una aproximación multidisciplinar. *European Journal of Human Movement* 31:77.

Morton, S., Otto, C., King, J., Perry, D., Crisp, T., Maffulli, N., Morrissey, D. 2014. High volume image-guided Injections for patellar tendinopathy: a combined retrospective and prospective case series. *Muscles, Ligaments & Tendons Journal* 4(2):214–219.

Muneta, T., Koga, H., Ju, Y.J., Mochizuki, T., Sekiya, I. 2012. Hyaluronan injection therapy for athletic patients with patellar tendinopathy. *Journal of Orthopaedic Science* 17(4):425-431.

Park, B.H., Seo, J.H., Ko, M.H., Park, S.H. 2013. Reliability and validity of the Korean version VISA-P Questionnaire for patellar tendinopathy in adolescent elite volleyball athletes. *Annals of Rehabilitation Medicine* 37(5):698–705.

Pascarella, A., Alam, M., Pascarella, F., Latte, C., Di Salvatore, M.G., Maffulli, N. 2011. Arthroscopic management of chronic patellar tendinopathy. *American Journal of Sports Medicine* 39(9):1975–1983.

Physical Therapy: News from the foundation for Physical Therapy 2010. http://ptjournal.apta.org/content/90/11/1697.full Retrieved 12 September 2016.

Research Report Abstracts: Physiotherapy 2011. http://www.physiotherapyjournal.com/issue/S0031-9406(11)X0004-4 Retrieved 12 September 2016.

Ryan, M., Wong, A., Rabago, D., Lee, K., Taunton, J. 2011. Ultrasound-guided injections of hyperosmolar dextrose for overuse patellar tendinopathy: A pilot study. *British Journal of Sports Medicine* 45(12):972–977.

Smith, J., Sellon, J.L. 2014. Comparing PRP injections with ESWT for athletes with chronic patellar tendinopathy. *Clinical Journal of Sport Medicine: Official Journal of the Canadian Academy of Sport Medicine* 24(1):88–89.

Sunding, K., Willberg, L., Werner, S., Alfredson, H., Forssblad, M., Fahlström, M. 2015. Sclerosing injections and ultrasound-guided arthroscopic shaving for patellar tendinopathy: Good clinical results and decreased tendon thickness after surgery-a medium-term follow-up study. *Knee Surgery, Sports Traumatology, Arthroscopy* 23(8):2259–2268.

Van Ark, M., Van den Akker-Scheek, I., Meijer, L.T.B., Zwerver, J. 2013. An exercise-based physical therapy program for patients with patellar tendinopathy after platelet-rich plasma injection. *Physical Therapy in Sport* 14(2):124–130.

Van Duren, B., Pandit, H., Murray, D., Gill, H. 2015. Approximation of the functional kinematics of posterior stabilised total knee replacements using a two-dimensional sagittal plane patello-femoral model: Comparing model approximation to in vivo measurement. *Computer Methods in Biomechanics & Biomedical Engineering* 18(11):1191–1199.

Verrall, G., Schofield, S., Brustad, T. 2011. Chronic achilles tendinopathy treated with eccentric stretching program. *Foot & Ankle International* 32(9):843–849.

Willberg, L., Sunding, K., Forssblad, M., Fahlström, M., Alfredson, H. 2011. Sclerosing polidocanol injections or arthroscopic shaving to treat patellar tendinopathy/jumper’s knee? A randomised controlled study. *British Journal of Sports Medicine* 45(5):411–415.

Xu, H., Bloswick, D., Merryweather, A. 2015. An improved OpenSim gait model with multiple degrees of freedom knee joint and knee ligaments. *Computer Methods in Biomechanics & Biomedical Engineering* 18(11):1217–1224.

Kim, K.H., Kim, T.G. 2011. Effects of patellar tendinopathy on the marche-fente movement of female fencing fleuret players. *Korean Journal of Sport Science* 22(2):1875–1883.

#### Exclusion of articles because of irrelevancy based on abstract screening (*n* = 12)

Bond, R.P., Snyckers, C.H. 2010. Management of sports overuse injuries of the lower limb: An evidence-based review of the literature. *South African Orthopaedic Journal* 9(2):48–58.

De Carlo, M., Armstrong, B. 2010. Rehabilitation of the knee following sports injury. *Clinics in Sports Medicine* 29(1):81–106.

Dimnjaković, D., Bojanić, I., Smoljanović, T., Mahnik, A., Barbarić-Peraić, N. 2012. Eccentric exercises in the treatment of overuse injuries of the musculoskeletal system. *Liječnički Vjesnik* 134 (1–2):29–41.

Eerkes, K. 2012. Volleyball Injuries. *Current Sports Medicine Reports* 11(5):251–256

Giombini, A., Dragoni, S., Di Cesare, A., Di Cesare, M., Del Buono, A., Maffulli, N. 2013. Asymptomatic achilles, patellar, and quadriceps tendinopathy: A longitudinal clinical and ultrasonographic study in elite fencers. *Scandinavian Journal of Medicine & Science in Sports* 23(3):311–316.

Gordon, A.I., DiStefano, L.J., Denegar, C.R., Ragle, R.B., Norman, J.R.; Cheatham, S. 2014. College and professional women’s basketball players’ lower extremity injuries: A survey of career incidence. *International Journal of Athletic Therapy & Training* 19(5):25–33.

Hernandez-Sanchez, S., Hidalgo, M.D., Gomez, A. 2011. Cross-cultural adaptation of VISA-P Score for patellar tendinopathy in Spanish population. *Journal of Orthopaedic & Sports Physical Therapy* 41(8):581–591.

Kristensen, J., Franklyn-Miller, A. 2012. Resistance training in musculoskeletal rehabilitation: a systematic review. *British Journal of Sports Medicine* 46(10):719–726.

Rio, E., Kidgell, D., Moseley, G.L., Cook, J. 2015. Elevated corticospinal excitability in patellar tendinopathy compared with other anterior knee pain or no pain. *Scandinavian Journal of Medicine & Science in Sports* 26(9):1072–1079.

Schwartz, A., Watson, J.N., Hutchinson, M.R. 2015. Patellar Tendinopathy. *Sports Health* 7(5):415–420.

White, T., Clapis, P. 2010. Patellar tendonitis (jumper’s knee) rehabilitation exercises. *Computerized Registration System – Sports Medicine Advisor* 1.

Young, M.A., Cook, J.L., Purdam, C.R., Kiss, Z.S., Alfredson, H. 2010. Patellar tendon injury (jumper’s knee) rehabilitation exercises: References. *Computerized Registration System – Sports Medicine Advisor* 1.

#### Exclusion of articles because of irrelevancy based on full-text screening (*n* = 17)

Couppé, C., Kongsgaard, M., Aagaard, P., Vinther, A., Boesen, M., Kjær, M., Magnusson, S. P. 2013. Differences in tendon properties in elite badminton players with or without patellar tendinopathy. *Scandinavian Journal of Medicine & Science in Sports* 23(2):89–95.

Culvenor, A.G., Cook, J.L., Warden, S.J., Crossley, K.M. 2011. Infrapatellar fat pad size, but not patellar alignment, is associated with patellar tendinopathy. *Scandinavian Journal of Medicine & Science in Sports* 21(6):405–411.

Dimitrios, S., 2015. There is lack of evidence to support the effectiveness of therapeutic ultrasound in the management of patellar tendinopathy. *Physical Therapy Reviews* 20(4):268–269.

Jain, N., Kemp, S., Hayward, P., Murray, D. J. 2012. Effect of pre-season screening for patella tendinopathy: The findings of a professional football club. *Muscles, Ligaments & Tendons Journal*:10.

James, L., Kelly, V., Beckman, E. 2014. Injury risk management plan for volleyball athletes. *Sports Medicine* 44(9):1185–1195.

Leal, C., Ramon, S., Furia, J., Fernandez, A., Romero, L., Hernandez-Sierra, L. 2015. Current concepts of shockwave therapy in chronic patellar tendinopathy. *International Journal of Surgery* 24(B):160–164.

Leporace, G., Pereira, G.R., Carmo, R.C.R., Silva, A.C., Cabral, R.P., Silva, N., Pasqualini, H.E.C., Batista, L.A. 2010. Specificity of the myoelectrical activity on the eccentric decline squat at 25 degrees and standard squat with different overloads. *Revista Brasileira de Medicina do Esporte* 16(3):205–209.

Malliaras, P., Cook, J., Purdam, C., Rio, E. 2015. Patellar tendinopathy: Clinical diagnosis, load management, and advice for challenging case presentations. The *Journal of Orthopaedic and Sports Physical Therapy* 45(11):887–898.

Mendonça, L. D., Bittencourt, N.F.N., Santos, T.R.T., Silva, A.A., Fonseca, S.T. 2011. Correlation of age, sex, body mass index and sports modality to patellar rotation in jumping athletes. *British Journal of Sports Medicine* 45(4):344.

Morton S., Morrissey D., Valle X., Chan O., Langberg H., Malliaras P. 2015. 2014. Equivalence of online and clinical administration of a patella tendinopathy risk factor and severity questionnaire. *Scandinavian Journal of Medicine & Science in Sports* 25(5):670–677.

Rio, E., Kidgell, D., Purdam, C., Gaida, J., Moseley, G.L., Pearce, A.J., Cook, J. 2015. Isometric exercise induces analgesia and reduces inhibition in patellar tendinopathy. *British Journal of Sports Medicine* 49(19):1277–1283.

Simpson, M., Smith, T.O. 2011. Quadriceps tendinopathy – A forgotten pathology for physiotherapists? A systematic review of the current evidence-base. *Physical Therapy Reviews* 16 (6):455–461.

Sorenson, S.C., Arya, S., Souza, R.B., Pollard, C.D., Salem, G.J., Kulig, K. 2010. Knee extensor dynamics in the volleyball approach jump: The influence of patellar tendinopathy. *Journal of Orthopeadic & Sports Physical Therapy* 40(9):568–576.

Wearing, S.C., Locke, S., Smeathers, J.E., Hooper, S.L. 2015. Tendinopathy alters cumulative transverse strain in the patellar tendon after exercise. *Medicine & Science in Sports & Exercise* 47(2):264–271.

Wilgen, C. P., Konopka, K. H., Keizer, D., Zwerver, J. 2013. Do patients with chronic patellar tendinopathy have an altered somatosensory profile? A quantitative sensory testing ( QST) study. *Scandinavian Journal of Medicine & Science in Sports* 23(2):149–155.

Yun, S., Jin, W., Park, Y.K., Kim, G., Yoon, S., Park, S., Lee, J., Park, J., Ryu, K. 2015. Increased signal intensity at the proximal patellar tendon: Correlation between MR imaging and histology in eight cadavers and clinical MR imaging studies. *European Radiology* 25(10):2976–2983.

Zhang, Z.J., Ng, G.Y., Lee, W.C., Fu, S.N. 2014. Changes in morphological and elastic properties of patellar tendon in athletes with unilateral patellar tendinopathy and their relationships with pain and functional disability. *PLoS One* 9(10):1–9.

#### Exclusion of articles because of poor methodological quality (*n* = 5)

Nagraba, L, Mitek, T., Stolarczyk, A., Lebiediew, A., Przymorski, T. 2010. Tendinopathy of the patellar ligament (jumper’s knee) – Diagnostics and treatment. *Arthroscopy and Joint Surgery* 6(3–4):19–23.

Pearson, S.J., Hussain, S.R. 2014. Region-specific tendon properties and patellar tendinopathy: A wider understanding. *Sports Medicine* 44(8):1101–1112.

Rodriguez-Merchan, E.C. 2013. The treatment of patellar tendinopathy. *Journal of Orthopaedic Traumatology* 14:77–81.

Rudavsky, A., Cook, J. 2014. Physiotherapy management of patellar tendinopathy (jumper’s knee). *Journal of Physiotherapy* 60(3):122–129.

Rutland, M., O’Connell, D., Brismee, J.M., Sizer, P., Apte, G., O’Connell, J. 2010. Evidence-supported rehabilitation of patellar tendinopathy. *North American Journal of Sports Physical Therapy* 5(3):166–178.

## References

[CIT0001] Da Cunha R.A., Dias A.N., Santos M.B. & Lopes A.D, 2012, ‘Comparative study of two protocols of eccentric exercise on knee pain and function in athletes with patellar tendinopathy: Randomised controlled study’, *Revista Brasileira de Medicina do Esporte* 18(3), 167–170. 10.1590/S1517-86922012000300006

[CIT0002] De Vries A., Zwerver J., Diercks R., Tak I., Van Berkel A., Van Cingel R. et al, 2015b, ‘Effect of patellar strap and sports tape on pain in patellar tendinopathy: A randomised controlled trial’, *Scandinavian Journal of Medicine and Science in Sports* 26(10), 1217–1224. 10.1111/sms.12556 26376953

[CIT0003] De Vries A.J., Van der Worp H., Diercks R.I., Van den Akker-Scheek I. & Zwerver J, 2015a, ‘Risk factors for patellar tendinopathy in volleyball and basketball players: A survey-based prospective cohort study’, *Scandinavian Journal of Medicine and Science in Sports* 25(5), 678–684. 10.1111/sms.12294 25091500

[CIT0004] Dimitrios S., Pantelis M. & Kalliopi S, 2012, ‘Comparing the effects of eccentric training with eccentric training and static stretching exercises in the treatment of patellar tendinopathy. A controlled clinical trial’, *Clinical Rehabilitation* 26(5), 423–430. 10.1177/0269215511411114 21856721

[CIT0005] Frizziero A., Trainito S., Oliva F., Aldini N.N., Masiero S. & Maffulli N, 2014, ‘The role of eccentric exercise in sport rehabilitation’, *British Medical Bulletin* 110(1), 47–75. 10.1093/bmb/ldu006 24736013

[CIT0006] Goldman R.B. & Lentz T.A, 2010, ‘The use of eccentric overloading exercise for the treatment of patellar tendinosis in an Olympic-style weightlifter: A case report’, *Orthopaedic Practice* 22(2:10), 76–82.

[CIT0007] Malliaras P., Barton C.J., Reeves N.D. & Langberg H, 2013, ‘Achilles and patellar tendinopathy loading programmes: A systematic review comparing clinical outcomes and identifying potential mechanisms for effectiveness’, *Sports Medicine* 43(4), 267–286. 10.1007/s40279-013-0019-z 23494258

[CIT0008] Mani-Babu S., Morrissey D., Waugh C., Screen H. & Barton C, 2015, ‘The effectiveness of extracorporeal shock wave therapy in lower limb tendinopathy: A systematic review’, *American Journal of Sports Medicine* 43(3), 752–761. 10.1177/0363546514531911 24817008

[CIT0009] McCreesh K.M., Riley S.J. & Crotty J.M, 2013, ‘Neovascularity in patellar tendinopathy and the response to eccentric training: A case report using Power Doppler ultrasound’, *Manual Therapy* 18(6), 602–605. 10.1016/j.math.2012.09.001 23022320

[CIT0010] Moher D., Liberati A., Tetzlaff J., Altmans D.G. & PRISMA Group, 2009, ‘Preferred reporting items for systematic reviews and meta-analyses: The PRISMA statement’, *Public Library of Science Medicine* 6(7), e1000097. 19621072 10.1371/journal.pmed.1000097 PMC2707599

[CIT0011] Romero-Rodriguez D., Gual G. & Tesch P.A, 2011, ‘Efficacy of an inertial resistance training paradigm in the treatment of patellar tendinopathy in athletes: A case-series study’, *Physical Therapy in Sport* 12(1), 43–48. 10.1016/j.ptsp.2010.10.003 21256449

[CIT0012] Saggini R., Di Stefano A., Galati V., Panelli E., Valeri M., Di Pancrazio L et al, 2012, ‘Long-term effectiveness of combined mechanotransduction treatment in jumper’s knee’, *European Journal of Inflammation* 10(3), 515–524.

[CIT0013] Samukawa M, 2011, ‘Management of patellar tendinosis in a freestyle mogul skier’, *International Journal of Athletic Therapy and Training* 16(2), 12–15. 10.1123/ijatt.16.2.12

[CIT0014] Silva R.S., Ferreira A.L.G., Nakagawa T.H., Santos J.M. & Serrão F.V, 2015, ‘Rehabilitation of patellar tendinopathy using hip extensor strengthening and landing-strategy modification: Case report with 6-month follow-up’, *Journal of Orthopaedic and Sports Physical Therapy* 45(11), 899–909. 10.2519/jospt.2015.6242 26390271

[CIT0015] Souza R.B., Arya S., Pollard C.D., Salem G. & Kulig K, 2010, ‘Patellar tendinopathy alters the distribution of lower extremity net joint movements during hopping’, *Journal of Applied Biomechanics* 26(3), 249–255. 10.1123/jab.26.3.249 20841615

[CIT0016] Toppi J., Fairley J., Cicuttini F.M., Cook J., Davis S.R., Bell R.J. et al, 2015, ‘Factors associated with magnetic resonance imaging defined patellar tendinopathy in community-based middle-aged women: A prospective cohort study’, *BMC Musculoskeletal Disorders* 16, 184–190. 10.1186/s12891-015-0645-8 26242763 PMC4526288

[CIT0017] Van der Worp H., Van Ark M., Roerink S., Pepping G.J., Van den Akker-Scheek I. & Zwerver J, 2011b, ‘Risk factors for patellar tendinopathy: A systematic review of the literature’, *British Journal of Sports Medicine* 45(5), 446–452. 10.1136/bjsm.2011.084079 21367808

[CIT0018] Van der Worp H., Van Ark M., Zwerver J. & Van den Akker-Scheek I, 2012, ‘Risk factors for patellar tendinopathy in basketball and volleyball players: A cross-sectional study’, *Scandinavian Journal of Medicine and Science in Sport* 22(6), 783–790. 10.1111/j.1600-0838.2011.01308.x 21496108

[CIT0019] Van der Worp H., Zwerver J., Kuijer P.P.F.M., Frings-Dresen M.H.W. & Van den Akker-Scheek I, 2011a, ‘The impact of physically demanding work of basketball and volleyball players on the risk for patellar tendinopathy and on work limitations’, *Journal of Back and Musculoskeletal Rehabilitation* 24(1), 49–55. 10.3233/BMR-2011-0274 21248400

[CIT0020] Van der Worp H., Zwerver J., Van den Akker-Scheek I. & Diercks R.L, 2011c, ‘The TOPSHOCK study: Effectiveness of radial shockwave therapy compared to focused shockwave therapy for treating patellar tendinopathy – Design of a randomised controlled trail’, *BMC Musculoskeletal Disorders* 12, 229–234. 10.1186/1471-2474-12-229 21989041 PMC3212818

[CIT0021] Zwerver J., Dekker F. & Pepping G.J, 2010, ‘Patient guided Piezo-electric Extracorporeal Shockwave Therapy as treatment for chronic severe patellar tendinopathy: A pilot study’, *Journal of Back and Musculoskeletal Rehabilitation* 23(3), 111–115. 10.3233/BMR-2010-0257 20858940

